# Involvement of Dopamine D1/D5 and D2 Receptors in Context-Dependent Extinction Learning and Memory Reinstatement

**DOI:** 10.3389/fnbeh.2015.00372

**Published:** 2016-01-21

**Authors:** Marion Agnès Emma André, Denise Manahan-Vaughan

**Affiliations:** ^1^Medical Faculty, Department of Neurophysiology, Ruhr University BochumBochum, Germany; ^2^International Graduate School for Neuroscience, Ruhr University BochumBochum, Germany

**Keywords:** extinction learning, dopamine, rodent, spatial learning, hippocampus, behavior

## Abstract

Dopamine contributes to the regulation of higher order information processing and executive control. It is important for memory consolidation processes, and for the adaptation of learned responses based on experience. In line with this, under aversive learning conditions, application of dopamine receptor antagonists prior to extinction result in enhanced memory reinstatement. Here, we investigated the contribution of the dopaminergic system to extinction and memory reinstatement (renewal) of an appetitive spatial learning task in rodents. Rats were trained for 3 days in a T-maze (context “A”) to associate a goal arm with a food reward, despite low reward probability (acquisition phase). On day 4, extinction learning (unrewarded) occurred, that was reinforced by a context change (“B”). On day 5, re-exposure to the (unrewarded) “A” context took place (renewal of context “A”, followed by extinction of context “A”). In control animals, significant extinction occurred on day 4, that was followed by an initial memory reinstatement (renewal) on day 5, that was, in turn, succeeded by extinction of renewal. Intracerebral treatment with a D1/D5-receptor antagonist prior to the extinction trials, elicited a potent enhancement of extinction in context “B”. By contrast, a D1/D5-agonist impaired renewal in context “A”. Extinction in the “A” context on day 5 was unaffected by the D1/D5-ligands. Treatment with a D2-receptor antagonist prior to extinction had no overall effect on extinction in context “B” or renewal in context “A”, although extinction of the renewal effect was impaired on day 5, compared to controls. Taken together, these data suggest that dopamine acting on the D1/D5-receptor modulates both acquisition and consolidation of context-dependent extinction. By contrast, the D2-receptor may contribute to context-independent aspects of this kind of extinction learning.

## Introduction

During extinction learning, conditioned responses become diminished during exposure to the conditioned stimulus (CS) in the absence of the unconditioned stimulus (US; Bouton, 2004; Myers and Davis, 2007). Extinction learning does not eliminate or erase the original memory, but rather mediates the creation of a new representation that allows the animal to ignore its behavioral responses to the previously learned conditioned stimuli (Rescorla, 2001). This process is reinforced by a change of context (Bouton, 2004), even under non-aversive (appetitive) conditions (Wiescholleck et al., 2014; André et al., 2015a,b). Reinstatement, or renewal, of the original conditioned response is typically reactivated upon re-exposure to the CS in the original context, or to conditions that are sufficiently dissimilar to the extinction context (Rachman, 1989; Bouton, 2004; Craske et al., 2008). Neuromodulators such as dopamine play a crucial role in memory processes and regulate synaptic information storage mechanisms such as synaptic plasticity (Hansen and Manahan-Vaughan, 2014). Dopamine is particularly important for the emotional weighting of experiences, but also for memory consolidation (Huang and Kandel, 1995; Bissière et al., 2003; Sajikumar and Frey, 2004; Lisman et al., 2011). It regulates cue-dependent fear conditioning (Fadok et al., 2010), and the consolidation of extinction of fear memory (Holtzman-Assif et al., 2010). This may be related to the role of the dopaminergic system in processing prediction errors as a component of associative learning (Schultz, 2006), or to the role of dopamine in reinforcing encoding of aversive experience. Less is known about the role of dopamine in appetitive context-dependent extinction learning processes that are supported by the hippocampus, and the precise role of dopamine receptor subtypes in this phenomenon is unclear.

The vast majority of studies on the role of dopamine in extinction and renewal have been conducted with regard to fear extinction (Abraham et al., 2014). Where appetitive processes have been explored, the focus has been on addiction (Di Chiara, 2002), rather than extinction of more benign appetitive processes such as the coupling of food-seeking behavior to specific non-aversive contexts. In the areas of fear memory and drug addiction, it is believed that the dopamine reward circuitry influences the encoding of the original aversive or appetitive experience (Lauzon et al., 2013) and extinction learning with regard to these experiences (Schultz and Dickinson, 2000). Strikingly, infusion of Levodopa (L-DOPA) a dopamine precursor, strongly promotes extinction regardless of the context and prevents fear memory from re-emerging (Haaker et al., 2013).

Recently, we reported that neurotransmitter receptor manipulations that are known to directly influence hippocampal synaptic plasticity and hippocampus-dependent learning, also modulate context-dependent extinction learning (André et al., 2015a,b). It has also been shown that the hippocampus contributes to context-dependent extinction learning and renewal of fear memory (Good and Honey, 1991; Ji and Maren, 2005; Hobin et al., 2006; de Carvalho Myskiw et al., 2014; Portugal et al., 2014; Tan et al., 2014). Dopamine receptors are expressed throughout the brain within regions that are key for the encoding and retrieval of long-term memory, such as the hippocampus, as well as in reward circuitry structures (Mansour and Watson, 1995). Whereas dopamine D1/D5-receptors are critically required for multiple forms of hippocampal synaptic plasticity, D2-receptors appear to contribute less to hippocampal plasticity processes, serving rather, to regulate hippocampal basal excitability tonus (Hansen and Manahan-Vaughan, 2014). Both dopamine D1/D5 (Hikind and Maroun, 2008) and dopamine D2-receptors (Mueller et al., 2010) have been implicated in extinction learning, however. Whereas dopamine D2-receptors positively couple to adenylyl cyclase, dopamine D1/D5-receptors are negatively coupled to this enzyme (Hansen and Manahan-Vaughan, 2014). Intuitively, one would expect that this means that dopamine D1/D5 and D2-receptors mediate opposing excitatory and inhibitory cellular responses, but whether this occurs or not depends on the relative activation of these receptors in specific brain regions, and the kind of associative learning event to be stored or retrieved. Evidence exists that dopamine D1/D5-receptors support fear acquisition and extinction (Inoue et al., 2000; El-Ghundi et al., 2001). Whether dopamine D2-receptors support these processes is less clear. Transgenic mice that lack dopamine D2-receptors exhibit a normal fear-potentiated startle response (Fadok et al., 2010). Others have shown that fear extinction is impaired (Holtzman-Assif et al., 2010; Mueller et al., 2010) or enhanced (Ponnusamy et al., 2005) by D2-receptor antagonists. The role of dopamine D1/D5 and D2-receptors in non-aversive appetitive extinction learning is also unclear.

In this study, we explored the role of dopamine D1/D5 and dopamine D2-receptors in extinction and renewal of a context-dependent appetitive spatial learning task. We observed that whereas dopamine D1/D5-receptor manipulation altered context-dependent extinction learning, dopamine D2-receptor manipulation affected context-independent aspects of this form of extinction learning. These data suggest, that with regard to appetitive experience, a differentiation may exist as to the contribution of dopamine D1/D5 and dopamine D2-receptors to key components of extinction learning that is supported by a context-change.

## Materials and Methods

The present study was carried out in accordance with the European Communities Council Directive of September 22nd, 2010 (2010/63/EU) for care of laboratory animals. All experiments were performed according to the guidelines of the German Animal Protection Law and were approved by the North Rhine-Westphalia State Authority (Bezirksamt, Arnsberg). All efforts were made to reduce the number of animals used.

### Animals

Male Wistar rats (7–8 weeks old) underwent implantation of guide cannulae, whilst under anesthesia (52 mg/kg sodium pentobarbital via intraperitoneal (i.p.) injection), as described previously (Manahan-Vaughan, 1997). One cannula was implanted into the lateral cerebral ventricle of each hemisphere (0.5 mm posterior to bregma, 1.6 mm lateral to the midline; size: 5.6 mm length, 0.8 mm diameter, 4.5 mm depth).

Animals were allowed 2 weeks to recover, before any behavioral experiment took place. They were housed singly and maintained on a 12 h light/12 h dark cycle with food and water *ad libitum*.

Two days prior to behavioral training, animal weight was determined and food availability was reduced to achieve 85% of this predetermined body weight. The animal’s weight was subsequently maintained at this level until the end of the experiment. Before beginning the experiment, animals were handled individually for 20 min per day.

### T-Maze and Extinction Task

Experiments were conducted in a T-maze that was composed of a starting box (25 × 20 cm) that was separated from the main corridor (100 × 20 cm) by a sliding door and two side corridors (40 × 10 cm) positioned perpendicular to the other end of the main corridor. The maze design and the protocol followed was as described previously (Wiescholleck et al., 2014; André et al., 2015a,b). The context of the maze was changed by exchanging the plastic floor of the maze (zebra stripes, checkered patterns, or geometric lines), odor cues that were placed at the end of the goal arms, and exchanging the extra-maze cue cards that were placed 40 cm above the end of the main corridor (Wiescholleck et al., 2014).

Every day, rats engaged in a learning session that comprised 20 consecutive trials, that were split into two data blocks (1st ten, 2nd ten trials), for analysis purposes (see below, and Wiescholleck et al., 2014; André et al., 2015a,b). The trial commenced when the door to the starting box was opened and the animal entered the maze. It ended when the animal entered a goal arm of the T-maze or when a predetermined time-limit (30 s to 2 min) had elapsed without arm entry (see below). Animals learned to search for a food pellet (Dustless Precision Pellets 45 mg, BioServ, USA) that was placed at the end of a predetermined goal arm. From day 1 through three reward probability was decreased from 100 to 25%. In conjunction with this, the time allowed to reach the arm was decreased in a stepwise manner from 2 min to 30 s. Learning criterion was reached when the animal successfully entered the correct arm on 8 of the last 10 trials of a 20 trial run. Failure to reach criterion by day 3 resulted in exclusion if the animal from subsequent trials (days 4 and 5). Its data from days 1–3 were not integrated into the data analysis for the study.

On day 4, extinction learning was assessed, whereby the animals explored the T-maze for 20 trials, during which time no reward was given (absence of the US). Here, the context was changed (novel floor, novel odors, novel cue cards). On day 5, renewal (RN) was assessed by re-introducing the animal to the original T-maze context (context “A”) for 20 trials with no food reward. Typically, animals respond to re-exposure to the “A” context by showing renewal in the 1st 10 trials followed by extinction in the 2nd set of 10 trials (resulting from the realization that no food reward is provided; Wiescholleck et al., 2014).

### Analysis of Decision Time

To assess choice confidence we measured the time taken by the animal to move from the departure area in the T-Maze to its arm of choice (Wiescholleck et al., 2014). As the confidence of the animal increases during the acquisition of the task, decision-time declines (Luce, 1986; Avila and Lin, 2014; Wiescholleck et al., 2014). We assessed this for every choice (not just correct choices) in order to determine the confidence of the animal in knowing which arm to enter.

### Pharmacological Treatment

All compounds were applied via a cannula that had been implanted into the lateral cerebral ventricle (see “Animals” Section). The D1/D5-receptor antagonist SCH 23390 (Tocris, Ellisville, MO, USA) was applied at a dose of 5.94 μg/μl. The D1/D5-receptor agonist, Chloro-PB (Sigma Aldrich St.Louis, MO, USA), was given at a dose of 8.33 μg/μl. The D2-like receptor antagonist, (*S*)-(–)-3-bromo-*N*-[(1-ethyl-2-pyrrolidinyl)methyl]-2,6-dimethoxybenzamide (remoxipride), (Tocris, Ellisville, MO, USA), was administered at a dose of 10 μg/μl. These doses were chosen because they are effective in preventing hippocampal synaptic plasticity (Kulla and Manahan-Vaughan, 2000; Manahan-Vaughan and Kulla, 2003; Lemon and Manahan-Vaughan, 2006; Wiescholleck and Manahan-Vaughan, 2014). All compounds were dissolved in double-distilled water and given in an injection volume of 5 μl. Drugs were applied via the guide cannula at a rate of 1 μl/min and given 30 min prior to the commencement of the extinction learning trials on day 4.

At the doses used, the compounds elicited no general changes in behavioral state, such as state-dependent effects. These properties had been assessed as part of previous studies (Kulla and Manahan-Vaughan, 2000; Manahan-Vaughan and Kulla, 2003; Lemon and Manahan-Vaughan, 2006). To additionally verify this, we assessed locomotion (in m/s) from the time of exit from the start box to the end of the main arm (100 cm) for all trials of each animal on day 4, after treatment with a dopamine ligand or vehicle. In addition we assessed stereotypy in the form of head-weaving (total number) for the entire duration of all 20 trials on day 4.

### Data Analysis

Correct answers were defined as trials in which the animal moved directly to the predetermined goal arm. For analysis purposes, each 20 trial session was divided into two sets of 10 trials (first 10 and last 10 trials). The time taken to reach the end of the first arm visited was calculated for each trial. To analyze decision time, the time required to move from the departure box in the T-Maze to the first chosen arm was recorded for each trial, and data were segregated into four sets of five trials for each day, of which the times were averaged (Wiescholleck et al., 2014). Extinction learning effects were assessed by comparing animal performance during the first, or second, set of trials on day 4 with performance during the second set of trials on day 3. Renewal effects were assessed by comparing animal performance during the first set of trials on day 5 with performance during the second set of trials on day 4. To examine if renewal performance was equivalent to learning performance at the end of the acquisition training (extinction efficacy), animal performance during the first set of trials on day 5 with performance during the second set of trials on day 3.

Data were analyzed using analysis of variance (ANOVA) with repeated-measures including two within-subject factors (Day and Session) and two between-group factors (Treatment and Experimental Design) to assess for differences between control and propranolol-treated animals. Differences between trial blocks or between trials days of a specific group (control or ligand-treated animals) were assessed using Bonferroni *post hoc* tests. Except where “ANOVA” is mentioned explicitly, all *p* values in the results section correspond to values determined from the Bonferroni test. The level of significance was set at *p* < 0.05.

## Results

### Context-Dependent Extinction is Enhanced by Antagonism of Dopamine D1/D5-Receptors. Renewal is Unaffected

**Figure 1:**
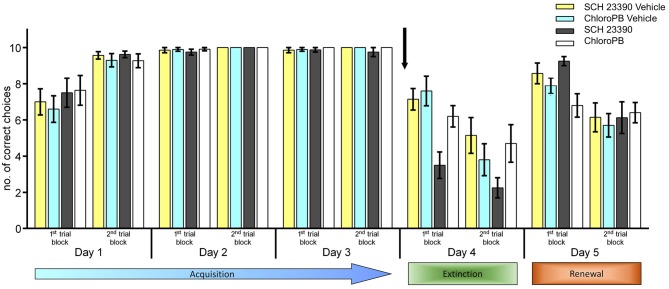
**Antagonism of dopamine D1/D5-receptors enhances extinction, but does not affect renewal.** Agonist activation of dopamine D1/D5-receptors has no effect on context–dependent extinction, but impairs renewal. Animals participated in 20 trials per day. Bar charts represent the number of correct arm choices in the first and second set of 10 trials on each test day. Three days of acquisition training (day 1–5), in context “A” were followed by extinction learning in a new context (day 4, context “B”) and re-exposure to the original context (context “A”) on day 5. Extinction of the learned conditioned stimulus (CS)-unconditioned stimulus (US) response occurred in the “A” context in control animals on day 5 (second 10 trials). No food was available on days 4 and 5. The arrow signifies the time of antagonist/vehicle-injection. The vehicle data for the antagonist group are labelled as “SCH 23390 vehicle” (yellow bars) and for the agonist group are labelled as “ChloroPB vehicle” (blue bars) Treatment of the animals with the dopamine D1/D5-receptor antagonist, SCH 23390 (dark gray bars), prior to the extinction learning trials on day 4 resulted in a significant enhancement of extinction (in the “B” context) compared to vehicle-treated controls (yellow bars). On day 5, renewal in context “A” was equivalent in both treatment groups (first 10 trials). Extinction of the CS-US response that had been learned in context “A” (2nd set of trials on day 5) was also equivalent in both treatment groups. Animals treated with the dopamine D1/D5-receptor agonist Chloro-PB (white bars) prior to exposure to the “B” context on day 4 showed significant extinction was evident by the 2nd set of 10 trials on day 4, that was not different from controls (blue bars). Upon returning to the same context on day 5, renewal of the conditioned behavior occurred in control animals (first 10 trials), whereas renewal was impaired in animals that had been treated on day 4 with the agonist. Extinction of the CS-US response that had been learned in context “A” (2nd set of trials on day 5) was equivalent in both treatment groups.

In the first 3 days of acquisition training, the animals successfully met the learning criterion. Thus, by the last 10 trials of day 3, animals made at least 8 out of 10 possible correct goal arm choices despite the reward probability having been reduced to 25% at this stage of acquisition training. A significant increase in correct choices was apparent between day 1 and day 2 (Figure 1; within-subject ANOVA: for animals subsequently treated with vehicle, *F*_(1,6)_ = 14.427; *p* = 0.009, *n* = 7; for animals subsequently treated with a dopamine D1 agonist, *F*_(1,7)_ = 9.215; *p* = 0.019, *n* = 8).

No significant difference was evident in performance within the first and second 10 trial blocks on day 3, signifying that the learning criterion had been achieved in both animal cohorts (Figure 1, *p* = 0.324). No significant difference in the animals’ learning behavior was found when the two animals cohorts were compared on days 1, 2 or 3. (Between-subject ANOVA: *F*_(1,13)_ = 0.029; *p* = 0.868).

Thirty minutes before commencing the extinction learning trials on day 4, either vehicle, or the dopamine D1/D5-receptor antagonist, SCH 23390 was applied. To facilitate extinction, the context of the environment was altered (context “B”: see “Materials and Methods” Section, and Wiescholleck et al., 2014). In vehicle-treated control animals, a significant attrition of correct choices became apparent that was significant in the last 10 trials of this session, when compared to the last 10 trials on day 3 (*p* < 0.001; Figure 1). Within-subject ANOVA confirmed that between day 3 and day 4, significant extinction learning occurred in vehicle-treated animals (*F*_(1,6)_ = 44.824; *p* < 0.001).

Treatment of the animals with the dopamine D1/D5-antagonist, 30 min prior to the extinction trials, significantly accelerated extinction (compared to controls, *p* < 0.001) and resulted in a better extinction effect overall (*F*_(1,7)_ = 124.096; *p* < 0.001; Figure 1).

On day 5, the animals were re-exposed to the context in which they had undergone acquisition training on days 1–3 (context “A”), with the exception that no food reward was available. Control animals and animals that had previously been treated with the dopamine D1/D5-antagonist responded immediately with renewal of the learned behavior (comparison of first 10 trials on day 5 with last 10 trials on day 4: *F*_(1,13)_ = 64.594; *p* < 0.001; Figure 1). During the last 10 trials of day 5, a significant deterioration of correct arm choices became apparent both animal groups (*p* < 0.001; Figure 1). This corresponds to extinction of the behavior learned in context “A”, as the animals realize that no reward can be expected. The profile of renewal and extinction in context “A” on day 5 was equivalent in vehicle-treated and antagonist-treated animals (*F*_(1,13)_ = 0.343; *p* = 0.568). These data suggest that the D1/D5 receptor may modulate context-dependent extinction. To clarify this, we examined the effects of agonist activation of D1/D5 receptors prior to extinction learning.

### Context-Dependent Extinction is not Affected by Agonism of Dopamine D1/D5-Receptors. Renewal is Impaired

Strikingly, animals that had been exposed to the dopamine D1/D5-receptor agonist, Chloro-PB (*n* = 8), exhibited extinction learning on day 4 (*F*_(1,17)_ = 13.68; *p* = 0.002: all trials day 4 vs. last 10 trials on day 3) that was equivalent to controls (*n* = 7; *F*_(1,17)_ = 0.646; *p* = 0.432; Figure 1). The treatment group showed impaired renewal on day 5, however (Figure 1). Here, the number of correct arm choices in the first 10 trials of day 5 was significantly fewer than during the last 10 trials of day 3 (*F*_(1,9)_ = 24.511; *p* < 0.001). In fact, performance was at the same level that had been apparent following successful extinction learning in these animals on day 4 (*F*_(1,9)_ = 2.295; *p* = 0.164, comparison of first 10 trials on day 5 with last 10 trials on day 4). No further deterioration of performance levels occurred during the second 10 trials on day 5 (*F*_(1,9)_ = 0.474; *p* = 0.509). Overall, a significant difference in choice behavior on days 4 and 5 was found when performance in vehicle-treated animals was compared with agonist-treated animals (*F*_(1,9)_ = 34.211; *p* < 0.01: all trials, day 4 vs. all trials, day 5).

### Context-Dependent Extinction and Renewal are Unaffected by Antagonism of Dopamine D2 Receptors. Context-Independent Extinction is Impaired

**Figure 2:**
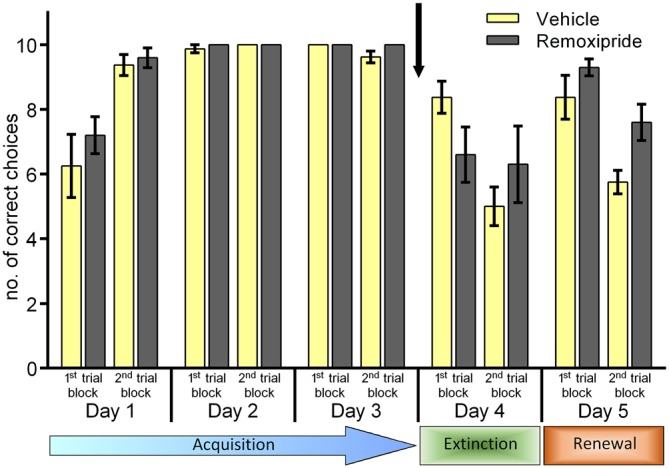
**Antagonism of dopamine D2-receptors has no effect on context-dependent extinction or renewal.** Extinction in the context-independent, context “A” is impaired. Treatment of the animals with the dopamine D2-receptor antagonist, Remoxipride, prior to the extinction learning trials on day 4 had no effect on extinction learning compared to vehicle-treated controls. Both groups exhibited significant extinction in the second set of 10 trials on day 4. On day 5, renewal in context “A” was equivalent in both treatment groups (first 10 trials). Extinction of the CS-US response that had been learned in context “A” (2nd set of trials on day 5) was impaired in the remoxipride-treatment group however.

We then tested the effects of a dopamine D2 receptor antagonist on context-dependent extinction learning (Figure 2). Animals were treated with remoxipride 30 min before starting the trials on day 4. Here also, we first verified that the animal cohorts that were subsequently treated with vehicle (*n* = 9) or remoxipride (*n* = 10) exhibited an equivalent learning performance during the acquisition days 1–3 (Figure 2; *F*_(1,16)_ = 1.441 ; *p* = 0.247). On day 4, following a change of T-maze context (context “B”) we assessed extinction learning. Here, although extinction was slightly better in the first 10 trials of day 4 in remoxipride-treated animals, overall no effect on animal behavior was apparent when performance in control and antagonist-treated animals was compared for the first and second trial blocks on day 4 (Figure 2; *F*_(1,17)_ = 0.646; *p* = 0.432). When the animals were returned to context “A” on day 5 no difference in their renewal performance was apparent, either (Figure 2; *F*_(1.17)_ = 0.284; *p* = 0.601, between-subject comparison of first 10 trials on day 5).

However, when performance within the antagonist-treated animals was assessed, a significant increase towards extinction-resistance in context “A” was observed (2nd trial block on day 5). Thus, extinction in context “A” was significantly poorer than that seen in vehicle-treated animals (Figure 2; *F*_(1,17)_ = 6.608; *p* = 0.02, between-subject comparison of last 10 trials on day 5).

### Antagonism of Dopamine D1/D5-Receptors Increases Decision-Time During Context-Dependent Extinction

**Figure 3:**
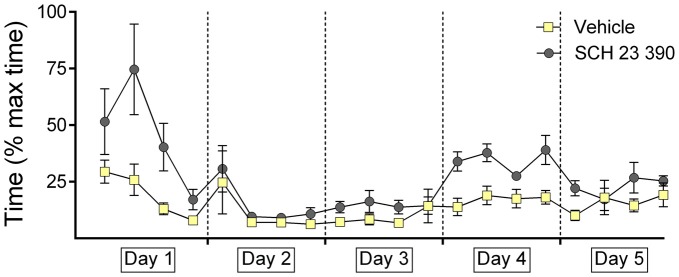
**Antagonism of dopamine D1/D5 impairs decision-time during context-dependent extinction learning.** The graph represents the amount of time that was needed to reach the end of an arm (both correct and incorrect choices) after door opening. For each day the time for five contiguous trials was averaged (i.e., four time-points per day are shown). Decision times recorded in animals that were treated with the dopamine D1/D5-receptor antagonist, SCH23390, or vehicle are shown. The vehicle or antagonist solution was injected 30 min prior to extinction learning in day 4. During learning of the task, the time required to reach the end of an arm continuously decreased in conjunction with a steady improvement in correct answers, until a basal level of correct answers was reached on day 3. During the extinction and renewal trials, the decision-time increased in parallel with the decrease of correct choices. The dopamine receptor antagonist significantly decelerated decision time during extinction learning on day 4. No performance differences were noted in drug or vehicle groups on day 5.

We have reported in the past that a gradual improvement in time to enter the first arm becomes evident as the animals acquire the task and become more confident as to the arm choice they should make (Wiescholleck et al., 2014). During extinction learning, decision-time increases once more in association with a decrease in the number of correct arm choices (Wiescholleck et al., 2014). The same performance profile was observed in the current study in vehicle—treated animals (Figure 3), whereby no performance differences were evident between the treatment groups on days 2 and 3 (*F*_(1.538,20)_ = 0.187; *p* = 0.774).

On day 4 (extinction learning), an increase in decision-time became evident, as the animals lost confidence in their choices (no arm was rewarded; *p* < 0.001). This was less apparent on day 5 (*p* = 1).

The animal cohort that was subsequently treated with SCH 23390 exhibited poorer decision times on day 1 of the study compared to controls, but by day 2, and extending through day 3 performance was equivalent in both animal cohorts (Figure 3). A clear learning effect occurred on days 1 through 3 (within-subject ANOVA: *F*_(1,9)_ = 14.961, *p* = 0.004).

A significant increase in decision-time was evident during extinction learning (in the presence of the antagonist) on day 4 (Figure 3; *p* < 0.001). Furthermore, the decision-time increase was significantly different to that observed in controls 3 (*F*_(1,13)_ = 31.992; *p* < 0.001).

On day 5, decision times were equivalent in both cohorts (Figure 3; *F*_(1,13)_ = 2.697; *p* = 0.125).

### Agonist Activation of D1/D5 Receptors Increases Decision-Time During Renewal and Subsequent Extinction of Context “A”

**Figure 4:**
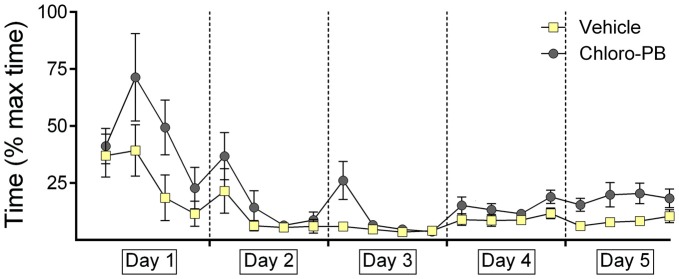
**Agonist activation of dopamine D1/D5 receptors impairs decision-time during renewal of the learned response.** The graphs represent the amount of time that was needed to reach the end of an arm (both correct and incorrect choices) after door opening. For each day the time for five contiguous trials was averaged (i.e., four time-points per day are shown). Decision times recorded in animals that were treated with the dopamine D1/D5-receptor agonist, Chloro-PB, or vehicle are shown. The vehicle, or agonist, solution was injected 30 min prior to extinction learning in day 4. The dopamine receptor agonist significantly impaired decision times during performance trials on day 5. No performance differences were noted in drug or vehicle groups on day 4.

Animals that were treated with the D1/D5-agonist Chloro-PB on day 4 showed equivalent decision times in the period of days 1–4 (Figure 4; Days 1–3: *F*_(2.803,50.459)_ = 1.899; *p* = 0.145; Day 4: *p* = 0.085).

On day 5, a significant increases in decision-time was evident in agonist-treated animals (*p* = 0.016; Figure 4). This aligns with our observation that renewal was impaired in the Chloro-PB group on day 5.

### Antagonism of Dopamine D2-Receptors has no Effect on Decision-Times

**Figure 5:**
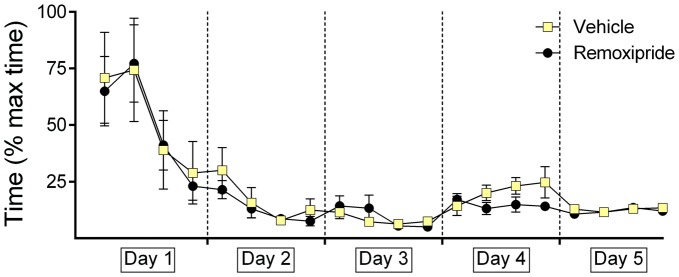
**Antagonism of dopamine D2-receptors has no effect on decision-time during context-dependent extinction learning, or renewal of the learned response.** The graphs represent the amount of time that was needed to reach the end of an arm (both correct and incorrect choices) after door opening. For each day the time for five contiguous trials was averaged (i.e., four time-points per day are shown). Decision times recorded in animals that were treated with the dopamine D2-receptor antagonist, Remoxipride, or vehicle are shown. The vehicle or antagonist solution was injected 30 min prior to extinction learning in day 4. The dopamine D2-receptor antagonist had no significant effect on decision time during extinction learning on day 4, or renewal on day 5.

The animal cohorts that were subsequently treated on day 4 with the D2-receptor antagonist, Remoxipride showed equivalent decision-times, as their vehicle-treated counterparts on days 1–3 (Figure 5). Performance on days 1 through 3 was equivalent in both groups (within-subject ANOVA: *F*_(1,16)_ = 0.079, *p* = 0.797). Although a tendency towards improved decision-time was evident on day 4, effects were not significant (Between-subject *F*_(1,17)_ = 0.037; *p* = 0.85).

Decision-times were also equivalent on both groups on day 5 (Between-subject *F*_(1,17)_ = 2.079; *p* = 0.168).

### The Dopamine Receptor Ligands had no Effect on Locomotion or Stereotypy

No significant differences in locomotion behavior were detected on day 4 after treatment with either dopamine receptor ligand or vehicle. In vehicle-treated animals (*n* = 10) locomotion speed was 0.62 ± 0.034 m/s, in ChloroPB –treated animals (*n* = 10) it was 0.64 ± 0.032 m/s (ANOVA: *F*_(1,18)_ = 0.196 *p* = 0.663), in SCH23390–treated animals (*n* = 8) it was 0.63 ± 0.031 m/s (ANOVA: *F*_(1,15)_ = 0.081 *p* = 0.78), and in Remoxipride–treated animals (*n* = 8) it was 0.66 ± 0.052 m/s (ANOVA: *F*_(1,16)_ = 0.539 *p* = 0.474; data not shown).

Similarly no significant effects with regard to stereotypy were observed. This was assessed as the number of head weavings conducted throughout all trials on day 4. Here, we observed an average of less than 1 head weaving during the total of 20 trials, for each of the animal groups tested (vehicle, ChloroPB, SCH23390, Remoxipride; data not shown).

## Discussion

In this study, we show that pharmacological antagonism of D1/D5-receptors enhances context-dependent extinction without affecting renewal or extinction of behavior in the original context. By contrast, agonist activation of D1/D5-receptors does not affect acquisition of extinction learning, but renewal of the conditioned behavior (context “A”) is impaired. Antagonism of D2-receptors neither has an effect on context-dependent extinction learning, nor does it affect renewal. Strikingly however, it increases resistance to extinction of the learned behavior in the original context. This suggests that under conditions where the fear circuitry cannot be expected to play a significant role in encoding and retrieval, dopamine D1/D5-receptors regulate context-dependent extinction, whereas dopamine D2-receptors may contribute to the learning of context-independent components of this form of extinction.

Our findings with regard to the involvement of dopamine D1/D5-receptors in the extinction of context-dependent appetitive spatial learning in rodents is in contrast to reports with regard to context-dependent fear extinction (Abraham et al., 2014). However, most studies that have addressed the role of these receptors in context-dependent fear extinction have done this by means of receptor antagonism, or transgenic animals that lack the receptor. Studies using a dopamine D1/D5 partial agonist demonstrated that extinction of fear-potentiated startle is impaired (Borowski and Kokkinidis, 1998), whereas prevention of dopamine/noradrenaline re-uptake enhances fear extinction (Abraham et al., 2012). We observed that blockade of D1/D5-receptors enhanced context-dependent extinction (in context “B”), and receptor activation impaired renewal of the behavior learned in the original “A” context. We propose that these differences can be explained by the brain circuitry that contributes to aversive learning and extinction, compared to non-aversive appetitive learning. In the case of fear learning, activation of the mesolimbic pathway and in particular the amygdala, prefrontal cortex and nucleus accumbens can be expected to predominate (Pezze and Feldon, 2004). In the case of appetitive learning, both the mesolimbic and the mesocortical pathways are involved (Abraham et al., 2014), whereby here, the role of the hippocampus in encoding context-dependent associations can be expected to be significant (Hansen and Manahan-Vaughan, 2014). Interestingly, activation of the locus coeruleus, that responds with noradrenaline release to context change (Bouret and Sara, 2005), and mediates heightened attention during appetitive extinction learning (André et al., 2015b), also results in modulation of ventral tegmental area (VTA) neurons (Grenhoff et al., 1993).

Central to both the mesolimbic and the mesocortical pathways is the VTA. Neurones of the dorsal VTA respond to reward-associated stimuli and their activity is suppressed by aversive stimuli, whereas neurons of the VTA increase their firing activity in response to negative or aversive stimuli (Brischoux et al., 2009). This suggests that a segregation occurs in the processing of reward-related and aversion-related information by the VTA. The ventral (ventromedial) VTA is reciprocally anatomically linked to the medial shell of the nucleus accumbens (Hasue and Shammah-Lagnado, 2002; Ikemoto, 2007), and aversive stimuli trigger dopamine release in this structure, as well as in the medial prefrontal cortex (Abercrombie et al., 1989; Kalivas and Duffy, 1995). Furthermore, dopamine receptor antagonists prevent fear learning if infused into the medial shell of the nucleus accumbens (Faure et al., 2008). The dorsal (dorsorostral) VTA, by contrast, projects predominantly to the amygdala, hippocampal formation and entorhinal region (Braak and Del Tredici, 2008). We are not disregarding the fact that the hippocampus is involved in the encoding of associative fear memory (Wen et al., 2015) and that the former circuit also recruits this structure (Abraham et al., 2014), however, the paradigm we implemented in the current study did not include a distinct aversive component, and therefore we assume that encoding of the associative learning experience was mediated by the latter projections from the VTA, thus possibly circumventing an intensive contribution of the nucleus accumbens.

When we applied a D1/D5-receptor antagonist we observed that extinction learning was immediately enhanced. Performance levels during the extinction trials were close to chance. Thus was in contrast to performance during the acquisition trials on day 1, when the animals first acquired the task. Here, however, the difference was that on day 1 in the first 10 trials all correct arms contained a reward, whereas during extinction learning none of the arms were rewarded: thus motivation levels can be expected to have been very different. Effects of the D1 antagonist on extinction learning were quite potent, but interestingly had no bearing on renewal performance one day after extinction learning had taken place. By contrast, D1/D5-receptor activation by means of an agonist had no ostensible effects on extinction learning in context “B”, but impaired subsequent renewal in context “A”. Taken together, these data suggest that in the absence of D1/D5-receptor activation, extinction learning in a new context is accelerated, although consolidation of this effect (and a resultant impact on renewal behavior) is not reinforced. By contrast, when D1/D5 receptors are activated, consolidation of extinction learning is reinforced and thus subsequent renewal of the original behavior (in the “A” context) is impaired. The lack of effect of the agonist on extinction learning can be explained by the likelihood that during the acquisition phase D1/D5 receptors may already be occupied by an adequate amount of dopamine, or D1/D5 receptors are not critically required for this component of extinction learning. An alternative, or perhaps complementary possibility is that the enhancement of extinction learning that was evident after D1/D5-receptor activation may have resulted from a modulation by the D1/D5-receptors of the saliency of the animal’s experience in the new (“B”) context (Hansen and Manahan-Vaughan, 2014). Thus, effects may not have derived solely, or exclusively, from an enhancement of consolidation, but rather from support of pattern separation through D1/D5-receptor activation.

A basal tonus of dopamine release has been described (Grace et al., 2007) that results in a homeostatic background activation of dopamine receptors. Phasic release of dopamine occurs when the VTA becomes activated by reward, aversive or error prediction events (Grace et al., 2007; Abraham et al., 2014). Given the fact that agonist activation of D1/D5-receptors had no ostensible impact on the extinction learning within the time frame of the T-maze trials, we assume that phasic activation may have been less important in the context-dependent extinction paradigm used in the present study. Thus, the antagonist may have prevented the action of tonically active D1/D5-receptors. As mentioned earlier, it is striking that extinction of the context-dependent appetitive task was enhanced by D1/D5-receptor antagonism, as studies with regard to fear extinction report that receptor antagonism impairs extinction (Inoue et al., 2000; El-Ghundi et al., 2001; Fadok et al., 2010). We think the difference relates to the anatomical circuitry mentioned above, and to the dopamine release patterns and brain structures triggered by these profoundly different behavioral experiences. Although the hippocampus is believed to be involved in both context-dependent aversive (Corcoran and Maren, 2001), and appetitive, extinction learning (André et al., 2015a,b), these processes are likely to be mediated by different cellular mechanisms: context-dependent fear memory triggers robust memory encoding through hippocampal long-term potentiation LTP (Whitlock et al., 2006), whereas non-aversive context-dependent learning triggers hippocampal long-term depression LTD (Manahan-Vaughan and Braunewell, 1999; Kemp and Manahan-Vaughan, 2004, 2007, 2008, 2012; Goh and Manahan-Vaughan, 2013). The antagonist treatment had no bearing on renewal. This is not surprising given the fact that acquisition of behavior in the “A” context had been consolidated before the antagonist was applied. Furthermore, and the application of the antagonist prior to extinction learning on day 4, might have prevented consolidation of the extinction learning experience in context “B”. In line with this, the impairment of renewal as a consequence of D1/D5-*agonist* treatment on day 4, suggests that consolidation of extinction learning, and/or the enhancement of the behavioral saliency of context “B” by D1/D5-receptor activation, served to firmly anchor the new memory created in context “B” and that this encoding impacted upon retrieval of the behavior previously learned in the “A” context. This observation is in line with many reports that support an important role for D1/D5-receptors in memory consolidation (Hikind and Maroun, 2008; Furini et al., 2014), in behavioral saliency (Hansen and Manahan-Vaughan, 2014), and in the long-term persistency of synaptic plasticity (Kulla and Manahan-Vaughan, 2000; Lemon and Manahan-Vaughan, 2006; Hansen and Manahan-Vaughan, 2014; Wiescholleck and Manahan-Vaughan, 2014).

We observed that antagonism of D2-receptors had no *ostensible* effect on context-dependent extinction learning, and also did not affect renewal in the “A” context. By contrast extinction learning within context “A” was impaired. Contradictory reports exist as to the involvement of this receptor in fear extinction (Ponnusamy et al., 2005; Fadok et al., 2010; Holtzman-Assif et al., 2010; Mueller et al., 2010). At the level of hippocampal information processing and this receptor plays a subordinate role: unlike the dopamine D1/D5-receptor, it does not critically contribute to the longevity and stability of LTP and LTD (Hansen and Manahan-Vaughan, 2014), rather activation of the D2-receptor serves to suppress synaptic excitability and lower basal tonus in the hippocampus (Manahan-Vaughan and Kulla, 2003). In line with this, a modulatory role for D2-receptors in spatial recognition memory (Léna et al., 2001) and passive avoidance learning (Sigala et al., 1997) have been reported. Dose-dependent beneficial and debilitatory effects of receptor antagonism for spatial reference memory have also been described (Setlow and McGaugh, 1999, 2000). This receptor may also be preferentially involved in the processing of aversive memories (Jocham et al., 2014; Wen et al., 2015).

It has been postulated, that at least at the level of the striatopallidal pathway, the D2-receptor may be important for learning flexibility (Yawata et al., 2012; Hatalova et al., 2014). Our findings suggest that extinction learning in context “B” may have recruited the support of information encoding in the hippocampus, to which the D2-receptor contributes little (Manahan-Vaughan and Kulla, 2003). Interestingly, the lack of extinction of the renewal effect on day 5, after application of the D2-receptor antagonist on day 4, suggests that blocking D2 receptors may nonetheless have affected learning flexibility. Thus, antagonism of D2-receptors may have affected the consolidation of context-dependent extinction learning, such that the memory of the original learned experience became more resilient. In this process, that reflects an impairment of extinction behavior in context “A”, other extra-hippocampal systems may predominate, to which activation of D2-receptors plays a significant part. In light of these findings it will be of interest to compare the involvement of D1/D5 and D2-receptors in context-independent forms of appetitive extinction learning.

## Concluding Remarks

Our data demonstrate that the dopamine D1/D5-receptor contributes to extinction learning of a context-dependent appetitive task by supporting extinction learning and by suppression of renewal. Antagonism of the receptor enhances extinction learning in a new context (in the absence of the US), but has no lasting impact on renewal or subsequent extinction on the original context. This suggests that tonic D1/D5-receptor activation modulates homeostatic processes whereby context-dependent information encoding is optimized. In line with this an interplay has been reported between D1/D5-receptors and the N-methyl-D-aspartate (NMDA) receptor that is critically required for multiple forms of hippocampal synaptic plasticity (Zweifel et al., 2009). Agonist activation of D1/D5-receptors had no ostensible impact during extinction learning *per se* (i.e., acquisition) but impaired subsequent renewal of the behavior learned in the “A” context. This is consistent with the likelihood that D1/D5-receptor promoted the consolidation of, and/or the behavioral saliency of the context change during, extinction learning that in turn, created interference for subsequent renewal behavior.

Under the conditions tested in our study, dopamine D2-receptors were not required for context-dependent extinction learning. An impairment of extinction of the conditioned behavior in the absence of the CS was evident, however, suggesting that antagonism of D2-receptors renders the original memory more resilient to extinction. Taken together, the findings of this study suggest that both D1/D5-receptors and D2-receptors modulate different components of extinction learning and renewal. Furthermore, the involvement of dopamine D1/D5 and D2-receptors in context-dependent appetitive extinction learning is distinct from their involvement in context-dependent fear extinction. We propose that this relates to the distinct neural circuitries that are activated by, and responsible for, the encoding of these different forms of behavioral experience.

## Conflict of Interest Statement

The authors declare that the research was conducted in the absence of any commercial or financial relationships that could be construed as a potential conflict of interest.
